# Perceptions of African Migrant Women Regarding Food Consumption During Pregnancy and the Postpartum Period in Australia: A Qualitative Study

**DOI:** 10.1111/jhn.70237

**Published:** 2026-03-29

**Authors:** Bolanle R. Olajide, Paige van der Pligt, Vidanka Vasilevski, Fiona H. McKay

**Affiliations:** ^1^ School of Health and Social Development, Institute for Health Transformation (IHT) Deakin University Burwood Victoria Australia; ^2^ Department of Allied Health, School of Health Sciences Swinburne University of Technology Hawthorn Victoria Australia; ^3^ School of Health and Social Development Deakin University Burwood Victoria Australia; ^4^ Department of Nutrition and Dietetics Western Health Footscray, Melbourne Victoria Australia; ^5^ School of Nursing & Midwifery, Centre for Quality and Patient Safety Research, Institute for Health Transformation Deakin University Burwood Victoria Australia; ^6^ Western Health St Albans Victoria Australia

**Keywords:** African migrants, Australia, food perceptions, food restrictions, postpartum, pregnancy, qualitative research

## Abstract

**Background:**

Women face challenges maintaining a healthy diet during pregnancy and the postpartum period. These challenges may be heightened for African migrant women who have cultural dietary preferences that can complicate food decision‐making. This study aimed to understand African women's food related perceptions after migrating to Australia.

**Methods:**

Eleven African migrant women who were either currently or had been pregnant in Australia were recruited. Qualitative photo‐elicitation interviews were conducted between November 2023 and March 2024. Participants shared photos representing foods they considered to be healthy and unhealthy during pregnancy and postpartum. A deductive qualitative approach to analysis using NVivo 14 was employed.

**Results:**

Two key themes were identified: 1) the perceptions of healthy foods during pregnancy and the postpartum period and 2) the perceptions of unhealthy foods during pregnancy and the postpartum period. Meals that were considered healthy for pregnancy were those that were balanced, homemade, and energy‐providing. Foods considered unhealthy were convenience and ultra‐processed foods.

**Conclusions:**

Participants' perceptions of healthy and unhealthy foods were not influenced by cultural dietary restrictions. Foods traditionally restricted during pregnancy in Africa were described as healthier choices. While women did maintain some cultural practices, they also described blending traditional African and Western foods in their diets.

## Summary

- African migrant women perceived healthy foods during pregnancy and the postpartum period as balanced, homemade, and energy‐providing meals, while convenience and ultra‐processed foods were consistently viewed as unhealthy.
- Women's perceptions of healthy and unhealthy foods were not shaped by culturally based pregnancy food restrictions; foods traditionally restricted in some African contexts were often described as healthy choices.
- Women described blending traditional African and Western foods after migration, indicating adaptive dietary practices rather than adherence to culturally restricted foods.

## 1 Introduction

During pregnancy, a healthy diet is important for the optimal growth and development of the fetus and to support vast physiological changes experienced by the mother [1]. A healthy diet during pregnancy should include an adequate intake of energy, macronutrients, and micronutrients to meet both maternal and fetal needs [2]. Consuming a healthy diet during this period is associated with a lower risk of gestational diabetes [3, 4], preterm delivery [5], and a healthy neonatal birth weight [6, 7]. In the postpartum period, a diet rich in essential nutrients is important for breast milk production [8] and supports infant growth, immunity [9, 10], and optimal metabolic development [8].

Women can struggle to maintain optimal nutrition during pregnancy and postpartum period [10, 11, 12]. This is related to insufficient knowledge about healthy eating [13, 14, 15], lack of time to prepare healthy meals [16, 17], financial constraints [13], and limited access to reputable nutrition guidance [18]. Cultural beliefs and traditions can also play a role, with some cultures encouraging dietary restrictions or consumption of specific foods during pregnancy and postpartum [8, 19, 20, 21].

Cultural dietary restrictions are defined as guidelines and customs that govern which food individuals are permitted or prohibited from consuming [22]. A recent review of research about migrant pregnant women from low ‐ and middle‐income countries found that many follow cultural dietary restrictions [21]. These cultural practices can influence diet quality during pregnancy and postpartum [21]. For example, pregnant women from Kenya and Ethiopia are often advised to restrict pineapple and eggs, due to a belief that they could be harmful [23, 24]. Postpartum women from Indonesia are often told to restrict fish as it is believed to cause unpleasant odour and bad tasting breastmilk [25]. Postpartum women from Ghana restrict bambara beans as they are believed to produce bad breast milk [26]. While some dietary restrictions are harmless, others may limit women′s nutritional intake and consequences for both the mother and infant.

Research shows that African women face barriers to healthy eating during pregnancy and postpartum after migration [27, 28]. Studies report limited access to familiar foods, misconceptions about food, and lack of social support as impacting women′s food choices and diet [14, 19, 27]. However, there is limited research examining how cultural dietary restrictions influence African migrant women's perceptions of a healthy diet during pregnancy and postpartum. Existing studies focus either on women's perceptions of a healthy diet during pregnancy or on their dietary transitions following migration [28, 29]. Most of these studies have been conducted in the United States of America (USA), with little evidence from countries with emerging African migrant populations, including the United Kingdom (UK) and Australia. Notably, no studies have explored African migrant women's perceptions of both healthy and unhealthy diets during pregnancy and postpartum. Addressing this gap is crucial, as pregnant migrant women often find it difficult to navigate between cultures and may face unique challenges in choosing a healthy diet in their new environment [27].

Over the past twenty years, Australia has seen a significant increase in African migration [30]. In 2003, there were approximately 232,390 individuals of African descent in Australia, a number that grew to 520,880 by 2023 [31]. Given this relatively recent growth in the African diaspora in Australia, this study aimed to understand African migrants' perceptions of healthy and unhealthy foods during pregnancy and the postpartum period. This is particularly important as African‐born women living in Australia are at higher risk of severe maternal outcomes such as very low birthweight, preterm birth, and severe postpartum hemorrhage compared to Australian‐born women [32, 33].

## 2 Methods

### 2.1 Study Design

This qualitative study employed semi‐structured interviews alongside a photo‐elicitation methodology. Photo‐elicitation methodology uses photographs taken by researchers or participants to prompt discussions through a semi‐structured interview [34, 35]. This encourages participants to actively reflect on their experiences while recalling memories and events. It also facilitates deep discussions between participant and researcher [36]. Photo‐elicitation has been used in previous research with pregnant migrant women [29].

### 2.2 Sample and Recruitment

**Figure 1:**
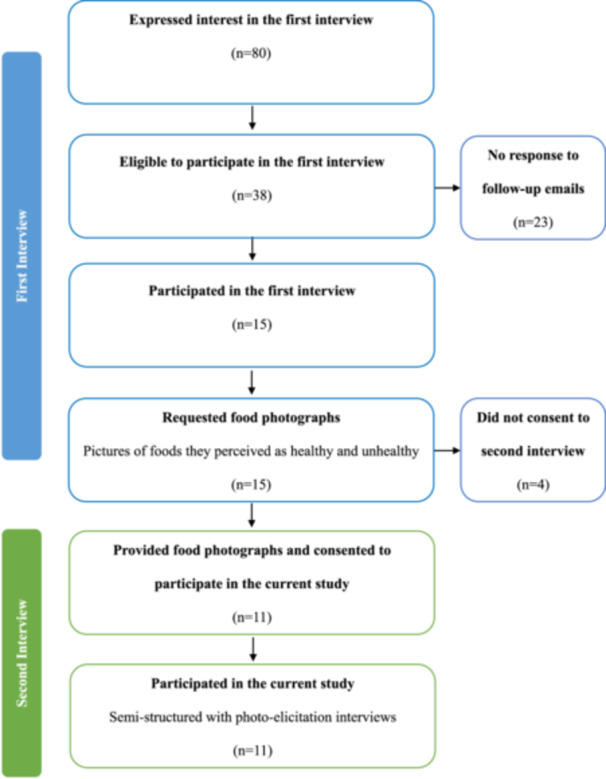
Process of data collection.

Participants in this study had previously taken part in a separate qualitative interview study [37] exploring cultural food practices in Australia (see Figure 1 for a detailed description). Participants in the current study were those who consented to take part in a second interview, which required them to take photographs to facilitate discussion. Eligible participants were women aged 18 and over, born in any African country and who were currently residing in Australia. All participants were either pregnant or had been pregnant in Australia within the past 5 years, owned a smartphone for taking photos, and were fluent in English.

Participants were recruited using purposive, convenience, and snowball sampling. Sampling involved reaching out to women through social media, women's groups, and the professional and personal networks of the research team. Snowball sampling involved participants informing other women about the study. Study details and researcher contact information were also shared via flyers placed in public spaces commonly visited by African communities.

### 2.3 Data Collection

Data were collected between November 2023 and March 2024. This study was approved by the Deakin University Human Research Ethics Committee (approval number: 2023‐331). All participants completed a short survey via Qualtrics to assess eligibility, and to provide consent and demographic information. The first author contacted all participants to arrange an interview. Following the first interview, participants were asked to use their smartphones to photograph foods they perceived to be healthy and unhealthy for consumption during pregnancy and postpartum over a week period. These photographs were used in discussions during the second interview.

Individual semi‐structured interviews were conducted via Zoom and guided by an interview guide (see supporting file 1). Interview questions were designed to enable discussion of photos in accordance with photo‐elicitation methodology [36]. Both the interview responses and the photos taken were collected as data sources.

During the interviews, individual participants' photos were displayed to enable discussion about the food. The first author conducted all interviews, which were audio‐recorded, lasted between 45 and 60 min, and were transcribed verbatim. Eleven women participated in the interviews. Although the initial plan was to recruit more participants, data saturation [38] was achieved after nine interviews, as subsequent interviews yielded repetitive information. The remaining two participants, who had already expressed interest, were still interviewed to honour their consent and this further confirmed data saturation. Participants were compensated for their time. Trustworthiness of the study was strengthened through member checking. Participants were given the option to review and validate their transcripts [39], only three participants chose to do so and requested no changes.

### 2.4 Data Analysis

A deductive qualitative analysis approach was employed to analyse the data based on the research objectives, organised under the following headings: perceptions of healthy and unhealthy foods during pregnancy, and perceptions of healthy and unhealthy foods during postpartum. This approach enabled a focused and structured analysis guided by specific research objectives. Deductive analysis supports the systematic exploration of qualitative data where the aim is to analyse participants' responses in relation to the research aims [40]. The first author conducted initial coding using NVivo 14. Although one author conducted the initial coding in NVivo, steps were taken to ensure the credibility and trustworthiness of the analysis. The research team met regularly throughout the analytic process to review, discuss, and refine the codes and themes. These collaborative discussions served as a form of peer debriefing, enabling the team to verify the coding decisions, challenge interpretations, and reach consensus on the final themes. This approach has been widely accepted in qualitative research to enhance rigour, even when coding is conducted by a single researcher. A preliminary coding framework was developed based on the study aim and reviewed by the research team. Photographs were not coded separately, rather they were incorporated into the analysis and served as reference points during participant narratives [36]. In this study, the photographs served as prompts to facilitate participant reflections and were not analysed as independent visual data sources, as the analytic focus was on women's perceptions. To preserve confidentiality, all participants were assigned a participant number. The study adhered to the Standards for Reporting Qualitative Research (SRQR), as outlined by O′Brien et al. [41] (see supporting file 2).

## 3 Results

Of the 15 women from the original study, 11 took part in the current study and provided between 3 and 10 photographs each. Participants were asked to select up to 5 photos they were willing to discuss during the interview. The sample consisted of married African migrant women from three African countries, see Table 1 for the participant demographic details.

**Table 1:** Demographic characteristics of 11 African migrant women who participated in photo‐elicitation interviews on their perceptions of food consumption during pregnancy and the postpartum period.

Demographic details	*n*
Country of birth	
Nigeria	8
Congo	2
Kenya	1
Ethnicity	
Yoruba	7
Others^a^	4
Age	
20–24	1
25–29	3
30–34	6
≥ 35	1
Parity	
Primiparous	6
Multiparous	5
Length of residence	
≤ 5 years	7
> 5 years	4
Level of education	
Bachelor's degree	9
Master's degree	2
Employment status	
Self employed	2
Unemployed	2
Employed	7

^a^ Black, Igbo, Bantu, and Luba.

Participants were asked to provide photographs of food they perceived to be both healthy and unhealthy for consumption during pregnancy and during the postpartum period. During the interviews, they were asked to explain why they considered these foods healthy or unhealthy. Deductive analysis of the transcripts resulted in two themes: 1) perceptions of healthy foods during pregnancy and the postpartum period and 2) perceptions of unhealthy foods during pregnancy and the postpartum period. Participants quotes are accompanied by participant number, country of birth, and length of residence in Australia.

### 3.1 Theme 1: Perceptions of Healthy Foods During Pregnancy and the Postpartum Period

Women shared their perceptions of foods they considered healthy during 1) pregnancy and the 2) postpartum period. This discussion encompassed their understanding of why a food might be considered ‘healthy’.

Eight women described the types of foods they considered healthy to eat during pregnancy. They explained that healthy foods were those that supported a balanced diet, provided energy, or were homemade. Women said that a ‘balanced diet′, including a combination of fruits, vegetables, and protein, was beneficial for both the growing baby and the mother. This sentiment was also reflected in a photograph one participant provided, which depicted a combination of spaghetti with vegetables and chicken as a balanced meal (see supporting file 3).

“If I am eating spag[hetti], I am putting a little bit of veggies just to have some fruit and veggies in it. I am putting my chicken. To me, that is a healthy meal because it is balanced… I think it helps with baby′s growth and development generally”.

(P3, Nigeria, 4 years)

Participants sought to incorporate food they considered ‘healthy′ for both the mother and the baby during pregnancy.

“Foods incorporated vegetable and meat, I believe that those were healthy options for myself as the mother and for my baby”.

(P7, Congo, 10 years)

Participants recognised the importance of specific nutrients during pregnancy and emphasised the role of homemade food in acquiring these nutrients. This sentiment was further illustrated in photographs provided by participants. One image, depicting a combination of brown beans, Irish potatoes, and amaranthus was specifically identified as both homemade and healthy (see supporting file 3).

“…. homemade food, it is well prepared, it has a source of fibre, we can see the protein [brown beans] …there is good source of fibre which are good for the baby”.

(P6, Nigeria, 3 years)

Participants described healthy foods that provide energy during pregnancy. Energy‐rich foods were considered carbohydrate‐rich sources such as rice, yam, and bread. Traditional “swallow” foods, which are dough‐like foods, such as poundo yam (made from either boiled yam tubers or flour mixed with water), eba (cassava flakes mixed with water), and amala (made from yam tuber or plantain flour) were also examples of energy‐dense foods.

“I have heard people say that just eat proteins and eat veggies when you are pregnant so that your baby will be healthy. But then where do we get energy from if we don′t eat food that has carbs [carbohydrate]. If we don′t eat rice… our local swallow meals. All of those things [foods listed] keeps us energetic while pregnant”.

(P3, Nigeria, 4 years)

While most participants (*n* = 10) considered carbohydrate‐rich sources important for pregnancy, one participant disagreed. This participant, who experienced gestational diabetes during pregnancy, said that these foods negatively affected her blood sugar levels. This sentiment was also reflected in the photographs she provided, demonstrating that foods such as rice and bread were considered unhealthy.

“…the quantity of the white rice or the starch or the food that contains carbs [carbohydrate]. I consider them unhealthy for me because they are not digesting so well. They are not working well with my metabolism”.

(P8, Nigeria, 7 years)

Most participants (*n* = 8) emphasised that consuming healthy food should not be confined to pregnancy alone.

“I feel that eating healthy is not something you do and stop. It is something that should be continuous even after pregnancy. That is why diet is a lifestyle. It is not like something you do and stop”.

(P2, Nigeria, 4 months)

Some women also described adapting their food choices in Australia in ways that shaped their perceptions of healthy foods. For example, one participant explained that although she traditionally paired akara (beans cake) with pap (corn pudding) in her home country, she substituted pap with oats in Australia due to availability.

“…If I am back home, I would not have taken food in picture number three (akara and oat). I would have had the akara… but I probably wouldn′t have had it with oats, I probably would have had that with pap”.

(P6, Nigeria, 3 years)

Other women described adopting foods from other cultures as healthy alternatives, reflecting changes in taste preferences and accessibility.

“There′s like Asian food, European food which are healthy… eventually my tastebud adapted here… Singapore noodles. And another thing is that I don′t think I would have been able to afford most of those things back home, but I do eat them here”.

(P8, Nigeria, 7 years)

The health impacts of consuming a healthy diet were discussed by four women (*n* = 4), indicating its support of blood sugar regulation and heart health. Two participants described the nutritional value of Okra for promoting health.

“Okra has like a lot of fibre, vitamin C and it is very healthy…it is very healthy in pregnancy because it helps with healthy hearts”.

(P10, Congo, 2 years)

Women discussed how the nutrient content of certain meals qualified them as healthy (see Table 2). Four participants indicated that traditional African dishes were healthy options during pregnancy. Although the remaining participants (*n* = 7) did not reflect this in their discussions, their photographs indicated a similar perception. These traditional foods included fufu (made from cassava tuber or flour), amala (made from yam flour or a mix of sorghum and plantain flour), eba (made from water and cassava flakes), ofada rice (brown rice), ugali (made from water and maize flour), and cassava leaf.

**Table 2:** Foods identified as ‘healthy’ by African migrant women during pregnancy and the postpartum period in photo‐elicitation interviews, along with illustrative quotes highlighting their perceived nutrient content.

Foods groups	Specific food	Participant quotes
Vegetables	Spinach, kale, avocado, amaranthus, coleslaw, egg plant, carrot, green peas, capsicum, lettuce, okra, vegetable salad, potatoes, cucumber, kumara, pumpkin, cassava leaf, and cabbage.	“Carrots is a good source of vitamin, the B vitamins. Cabbage too [is a source of] Vitamin C and K, and cucumbers, it has folic acid, vitamin B1, B2, and B3” (P9, Nigeria, 1 year). “… cassava leaf is good for improving breast milk and preventing constipation. It is a good source of fibre…It is very nutritious for mum” (P10, Congo, 2 years).
Fruits	Watermelon, apple, banana, pineapple, plantain, fruit juice.	“Taking fruits is healthy for myself as a young woman, [and] as a pregnant woman” (P2, Nigeria, 4 months).
Legumes and pulses	Beans, bean products (e.g., bean cakes) and lentils	“Beans is a good source of protein and fibre on its own” (P6, Nigeria, 3 years). “Lentil is protein…healthier than beans. I am not 100% sure, but to me that is what I think because I actually tried consumed [it during pregnancy] …” (P8, Nigeria, 7 years).
Grains	Bread, chapatti, rice, pasta, noodles, sweet corn, oats, eba (a swallow made from boiled water and cassava flakes), and amala (a swallow made from sorghum and unripe plantain flour).	“Brown rice is an excellent source of many nutrients like fibre, selenium, magnesium and the likes” (P9, Nigeria, 1 year).
Meat, fish, and poultry	Meat, (chicken, lamb, turkey, beef, goat, and cow offal stew, sausage), eggs, and fish.	“Meat is a source of protein…that is one place to get more protein” (P6, Nigeria, 3 years).
Beverages	Tea, chocolate milk drinks (i.e., Milo), milk (liquid full cream and powdered)	“I just know that it [milk] is a good source of protein and fibre” (P6, Nigeria, 3 years).
Snacks	Shawarma, steak sandwich	“It [shawarma] has more veggies in it and less cream… I just believe carrots in it is good for the eye, the skin and also brain development [for the baby] …” (P3, Nigeria, 4 years).

Two participants from Congo identified cassava leaf, a traditional African vegetable, as a healthy food. They emphasised that cassava leaf is rich in nutrients important for pregnancy and fetal development.

“Cassava leaf is one of our traditional vegetables. It is high in fibre and lots of folic [acid] … prevent stuff like spinal bifida because like the doctors make you take folic acid because of that, usually to help prevent spinal bifida in babies”.

(P7, Congo, 10 years)

One participant highlighted the healthiness of African dishes due to her familiarity with these foods and their benefits.

“My African food. I know it is healthy. I grew up eating it. I have been eating it since I have been aware of myself and I am still eating it and it is healthy for me also during pregnancy”.

(P5, Nigeria, 3 years)

While most participants (*n* = 10) considered traditional foods healthy, one participant perceived that ‘fufu′ (a swallow made from cassava tuber or flour) was unhealthy during pregnancy.

“Fufu is not too healthy…makes you very big during pregnancy… This may lead to high risk of like blood pressure or preeclampsia”.

(P8, Nigeria, 7 years)

Healthy foods during the postpartum period were described as meals that were balanced and filling. Most participants (*n* = 9) shared their understanding of healthy postpartum foods and emphasised the importance of a balanced diet to support recovery. They explained that postpartum healing required consuming essential nutrients for strength and energy.

“You just have to eat balanced diet after you put to bed [following birth], you want to eat well [healthy foods] so that you have more nutrient to your body…fit for your baby and you are strong enough”.

(P11, Nigeria, 5 years)

Women mentioned that a diet consisting of ‘filling foods′, which satisfied hunger quickly and were slow to digest was important. Breastfeeding mothers reported experiences of frequent hunger, requiring consumption of foods that provided lasting sustenance. Examples of filling foods included those high in fibre and protein, such as oats with milk, oatmeal with okra soup, and bean products.

“It fills you up [bean cakes with oats, milk, and sugar] and you know when you are breastfeeding, you tend to get hungry more so with this food… it fills you up and you don′t get hungry easily”.

(P5, Nigeria, 3 years)

Participants described foods that were perceived to increase breast‐milk production or aid in recovery after childbirth. Examples included pap (corn pudding), peanut butter soup, oatmeal, akara (bean cake), and hot okra soup. A photograph provided by one participant, which was described as ‘*traditional soup′* prepared with chicken and dried fish was perceived to support breastfeeding and postpartum recovery (see supporting file 3).

### 3.2 Theme 2: Perceptions of Unhealthy Foods During Pregnancy and the Postpartum Period

Participants also described foods they considered to be unhealthy during pregnancy and postpartum. These included foods with listeria risk, pre‐packed foods, high sugar foods, and drinks (see Table 3 for an overview of these foods).

**Table 3:** Foods identified as ‘unhealthy’ by African migrant women during pregnancy and the postpartum period in photo‐elicitation interviews, along with illustrative quotes highlighting their perceived reasons.

Foods considered unhealthy	Examples	Participant quotes
Foods with listeria risk	Cheese, fish, undercooked foods, unpasteurised dairy	“Cheese is not advisable that much during pregnancy… it could harm the baby or could harm you or make you sick…” (P4, Nigeria, 6 years).
Confectionery and pre‐packaged foods	Processed and canned foods, baked beans, chicken mayo (mayonnaise) and fried plantain, fried chips (potato chips), hash brown, take away foods, doughnuts, pizza, cakes, and meat pies	“…. when you fry the potatoes, I believe that all the nutrients go away…fatten [increase your bodyweight] … when [it is] time for giving birth, pushing will be difficult” (P1, Kenya, 7 years).
Sugar sweetened drinks	Hot chocolate drink, carbonated drinks (coke and sprite)	“…it has a lot of calories…having too much sugar too is not good especially when you′re pregnant… because of your health and that of the baby” (P9, Nigeria, 1 year). “Apart from the whole sugar…there are some other things that these drinks contain that we might not know about. Preservatives…” (P2, Nigeria, 4 months).
High carbohydrate foods	Fufu (a swallow made from cassava tuber or flour), rice, white bread	“Rice increases sugar levels once you are diabetic but if you are not diabetic in pregnancy, it has nothing to do…” (P8, Nigeria, 7 years)

Unhealthy foods during pregnancy were described as those high in calories and fat. Participants attempted to avoid these foods during pregnancy.

“Some of those confectioneries [for] example doughnuts, cakes, meat pies. Most of them…contain saturated fat…when eaten makes babies weight much… delivery might be an issue”.

(P2, Nigeria, 4 months)

Most participants (*n* = 6) perceived food from fast food restaurants to be unhealthy for pregnant women.

“Fried foods … I tried to avoid the KFC [Kentucky Fried Chicken] fried foods…[it] is not really a good option for a pregnant woman”.

(P11, Nigeria, 5 years)

Similarly, pre‐packed foods were identified as unhealthy, with items like baked beans and hash browns being identified. These foods were considered unhealthy due to their extensive processing. This sentiment was reflected in a photograph provided by one participant (see supporting file 3)

“Baked beans, definitely not healthy during pregnancy. It is a canned product, and it undergoes so many processes and everything so yeah, it is not a healthy meal to eat”.

(P6, Nigeria, 3 years)

In addition to pre‐packaged foods, one participant identified chicken mayonnaise as unhealthy for pregnant women, noting that it is processed and could increase the risk of nausea. (see supporting file 3).

“It is unhealthy because it is full of plantains [which] are fried in oil, and the chicken mayo [mayonnaise] is covered in mayo… mayo is not healthy because it is processed. Also, it might make you [pregnant woman] nauseous because of the mayo”.

(P10, Congo, 2 years)

Participants discussed the impact of consuming unhealthy foods, such as increased risk of weight gain, pregnancy complications, and long‐term health problems.

“Foods that are high in sugar or salt or unhealthy fat like pizza and fried foods. Those are really unhealthy to eat during pregnancy… [these foods] could also increase the risk of excess weight gain, hypertension, gestational diabetes, and could cause a long‐term implication for the child and for the mother”.

(P4, Nigeria, 6 years)

Participants described certain postpartum foods as unhealthy, particularly those that could affect the health of the newborn. Participants noted that a mother's diet during the postpartum period could impact on the infant's health, and having too much added sugar was considered unhealthy for breastfeeding mothers.

“So, consumption of sugar, too much of sugar. Maybe you want to take custard or tea, putting too much of sugar in your food… Baby takes this [sugar] from the breastmilk. So that is also unhealthy and not good for baby′s health”.

(P11, Nigeria, 5 years)

Few women (*n* = 3) also described that high sugar intake had immediate effects on their babies following breastfeeding.

“Someone who is breastfeeding, what you take in [will] reflect in the baby, so for me, if I am taking sugary things, my baby will keep pooing essentially because I am breastfeeding. The baby will keep pooing or have a running tummy or be constipated”.

(P3, Nigeria, 4 years)

## 4 Discussion

This study is the first to use a photo‐elicitation approach to explore African migrant women's perceptions of both healthy and unhealthy foods during pregnancy and postpartum in Australia. The use of photo‐elicitation enabled in‐depth discussions of food consumption during pregnancy and postpartum. This study contributes valuable insights into how African migrant women define healthy and unhealthy foods during pregnancy and postpartum period.

While previous studies with pregnant African women have explored barriers to maintaining a healthy diet during pregnancy [14, 19, 42], none have specifically explored African migrant women's perceptions of healthy and unhealthy foods during both pregnancy and postpartum. Understanding these perceptions is key to supporting healthy dietary behaviours across pregnancy and postpartum, especially amid cultural changes after migration. Foods considered healthy included both traditional African foods and those commonly consumed in Australia. This finding suggests that African migrant women may incorporate both local and traditional foods into their diets, challenging the assumption that migrant women face difficulties in adopting a healthy diet post‐migration [27, 29]. Foods considered unhealthy and therefore avoided during pregnancy were consistent with general dietary recommendations for pregnant women, rather than being culturally motivated.

This contrasts with the notable cultural dietary restrictions observed among African women during pregnancy and postpartum in certain African countries [23, 24, 26]. Previous research in Nigeria, Congo, and Kenya identified that foods such as eggs, meat, and bean products were restricted during pregnancy as they were considered unhealthy [23, 43, 44]. However, participants in the current study consumed these foods during pregnancy. This suggests that cultural dietary restrictions may not necessarily persist after migration for all individuals, possibly due to access to different food options or adaptation to new cultural contexts.

Participants in this study identified that consuming foods that were part of a balanced diet, offered energy, were homemade and provided sustenance were important for pregnancy and postpartum. A healthy diet consisted of fruits, vegetables, and proteins. A maternal diet rich in these food groups is associated with a reduced risk of hypertensive disorders during pregnancy [45]. For the unborn child, such foods are linked to a lower risk of allergic diseases [46], as well as the prevention of intrauterine growth restriction and low birth weight [10, 47]. This finding aligns with a study conducted among pregnant Sub‐Saharan African women (*n* = 11) in the USA, where participants described consuming foods from different groups to maintain a balanced diet [29]. While participants in this study depicted combinations of fruits, vegetables, and proteins in their photographs, it remains unclear whether they were aware of the specific quantities required during pregnancy or indeed if they were consuming sufficient quantities to meet nutritional guidelines. Since this aspect is beyond the scope of this study, further research is needed to assess participants' overall diet quality during pregnancy.

Healthy foods during the postpartum period were described by women in this study as ‘balanced′ and ‘filling,′ often accompanied by photographs of foods high in fibre or protein. This contrasts a study conducted in Ghana which reported that women were often encouraged to restrict fibre‐rich foods, such as sorrel leaves, due to belief that they reduce breastmilk production [26]. The differing interpretations in this study may reflect shifts in beliefs following migration or access to different postpartum norms within the Australian health system. This suggests that postpartum dietary perceptions among migrant women are shaped not only by cultural heritage but also by new information and practices encountered in the host country [37], highlighting the importance of culturally sensitive postpartum nutrition support.

Unhealthy diet during pregnancy and the postpartum period can increase the risk of poor health for both the mother and her child [48, 49, 50]. Women in this study described unhealthy foods as ultra‐processed foods with low nutritional value. While they identified foods that were indeed unhealthy, it is unknown whether this aligns with their actual consumption. This gap suggests that women may have a good conceptual understanding of unhealthy foods, but translating this knowledge into everyday food choices may be influenced by other factors such as convenience, affordability, or time constraints in preparing foods they described as healthy [51, 52]. Understanding this distinction is important for designing support that not only communicates nutrition messages but also addresses the practical barriers women face in applying them. Future research is needed to assess women's actual dietary intake during these periods.

Women's perceptions of what constituted healthy and unhealthy foods during pregnancy and postpartum were also shaped by broader contextual influences. Socioeconomic factors, including women's level of education, appeared to inform how they made sense of and evaluated healthy and unhealthy foods. Participants in this study were predominantly highly educated, which may have contributed to their ability to articulate clearer definitions of healthy and unhealthy foods. This aligns with previous research showing that individuals with higher levels of education often report more positive attitudes toward healthy eating and demonstrate higher dietary quality [53, 54]. While education is only one component of socioeconomic status, it may have contributed to how women assessed the healthfulness of foods due to higher levels of health literacy [55].

The limited availability and affordability of familiar African foods in Australia also appeared to influence women's perceptions of healthy and unhealthy foods. Consistent with evidence from African migrant women in the UK [52], USA [28], and Australia [37], participants described challenges accessing culturally familiar foods and noted that these ingredients were often expensive. For some women, this might have shaped how they viewed different food options. African foods were considered healthier but were difficult to obtain, whereas Western foods were more accessible but perceived as less healthy. These contextual constraints suggest that perceptions of healthfulness were influenced not only by beliefs but also by the realities of affordability and accessibility. Women also needed to adapt to a new food environment by substituting ingredients, modifying recipes, or relying on readily available foods. Recognising these influences is important for ensuring that nutrition support is both culturally relevant and feasible within women's everyday circumstances.

### 4.1 Strengths and Limitations

A key strength is the use of the photo‐elicitation method alongside semi‐structured interviews. This approach provided complementary insights, enhancing the credibility and validity of the findings. Incorporating photos during the interviews stimulated participants' reflections and encouraged them to describe their experiences.

Despite the strengths of this study, there are limitations that need to be considered. First, while the study explored women's perceptions of healthy and unhealthy foods during pregnancy and postpartum, it did not assess their actual dietary intake or nutritional status. This may introduce respondent bias, where the women told the researchers what they perceived to be socially desirable. Future research should evaluate women's dietary intakes during these periods to assess diet quality.

Second, this is a preliminary study of eleven women, and findings are not generalisable to all African migrant women. Third, eligibility criteria did not specify a minimum or maximum length of residence in Australia (time since migration) at recruitment. This may have introduced variation in dietary perceptions related to differences in settlement and acculturation. Length of residence was not restricted because recruitment within this population can be challenging, consistent with previous research [37] involving African migrant women in Australia. Applying additional criteria (e.g., minimum or maximum duration of residence) may have narrowed the eligible pool and reduced participation.

Fourth, the inclusion of one woman with gestational diabetes alongside women without pregnancy complications may have introduced differences in food perceptions. Gestational diabetes typically involves additional dietary advice, monitoring, and food‐related restrictions, which may shape how women interpret and describe “healthy” and “unhealthy” foods. Given the small sample, these potential differences could not be explored in depth. Finally, the eligibility criteria required participants to be English‐speaking and to own a smartphone, which may have excluded women with limited English proficiency or without access to digital devices, who may have different experiences or perceptions. Despite these limitations, the study provides valuable insights into the perceptions of African migrant women regarding food consumption during pregnancy and the postpartum period.

## 5 Conclusion

This study explored the perceptions of African migrant women regarding food consumption during pregnancy and the postpartum period. The findings revealed that participants continued to prefer their traditional African foods. Interestingly, cultural dietary restrictions commonly reported in certain African countries were not evident among this group. These findings should be interpreted with caution given the small and relatively homogeneous sample, and further research with more diverse groups of African migrant women is needed to confirm whether cultural dietary restrictions persist after migration. These findings suggest that healthcare providers should consider the flexibility and adaptability of cultural food practices when offering dietary guidance to women from diverse cultural backgrounds.

## Author Contributions

**Bolanle R. Olajide:** conceptualization, formal analysis, methodology, investigation, visualization, validation, writing – original draft, and writing – review and editing. **Paige van der Pligt:** conceptualization, formal analysis, methodology, supervision, validation, and writing – review and editing. **Vidanka Vasilevski:** conceptualization, formal analysis, methodology, supervision, validation, and writing – review and editing. **Fiona H. McKay:** conceptualization, formal analysis, methodology, supervision, validation, and writing – review and editing. All authors read and approved the final draft before submission.

## Ethics Statement

Ethics approval was obtained from the Deakin University Human Research Ethics Committee (high‐risk ethics), with approval number 2023‐331, granted on November 2, 2023.

## Consent

Before the interview, participants provided consent to participate in the research interview and to have their data published anonymously.

## Conflicts of Interest

The authors declare no conflicts of interest.

## Supporting information

Supporting file 1.

Supporting file 2.

Supporting file 3.

## Data Availability

The authors confirm that the data supporting the findings of this study are available within the article. Further inquiries can be directed at the corresponding author.

## References

[1] L. E. Forbes , J. E. Graham , C. Berglund , and R. C. Bell , “Dietary Change During Pregnancy and Women's Reasons for Change,” Nutrients 10, no. 8 (2018): 1032. 30096771 10.3390/nu10081032 PMC6115730

[2] World Health Organization . WHO recommendations on antenatal care for a positive pregnancy experience 2016. 28079998

[3] D. A. J. M. Schoenaker , G. D. Mishra , L. K. Callaway , and S. S. Soedamah‐Muthu , “The Role of Energy, Nutrients, Foods, and Dietary Patterns in the Development of Gestational Diabetes mellitus: A Systematic Review of Observational Studies,” Diabetes Care 39, no. 1 (2016): 16–23. 26696657 10.2337/dc15-0540

[4] M. Donazar‐Ezcurra , C. López‐del Burgo , and M. Bes‐Rastrollo , “Primary Prevention of Gestational Diabetes mellitus Through Nutritional Factors: A Systematic Review,” BMC Pregnancy and Childbirth 17 (2017): 30. 28086820 10.1186/s12884-016-1205-4 PMC5237148

[5] S. Abdollahi , S. Soltani , R. J. de Souza , S. C. Forbes , O. Toupchian , and A. Salehi‐Abargouei , “Associations Between Maternal Dietary Patterns and Perinatal Outcomes: A Systematic Review and Meta‐Analysis of Cohort Studies,” Advances in Nutrition 12, no. 4 (2021): 1332–1352. 33508080 10.1093/advances/nmaa156 PMC8321866

[6] M. Grandy , J. M. Snowden , J. Boone‐Heinonen , J. Q. Purnell , K. L. Thornburg , and N. E. Marshall , “Poorer Maternal Diet Quality and Increased Birth Weight,” Journal of Maternal‐Fetal & Neonatal Medicine: The Official Journal of the European Association of Perinatal Medicine, the Federation of Asia and Oceania Perinatal Societies, the International Society of Perinatal Obstetricians 31, no. 12 (2018): 1613–1619. 10.1080/14767058.2017.1322949 PMC5694379 28514885

[7] J. M. Martínez‐Galiano , C. Amezcua‐Prieto , I. Salcedo‐Bellido , G. González‐Mata , A. Bueno‐Cavanillas , and M. Delgado‐Rodríguez , “Maternal Dietary Consumption of Legumes, Vegetables and Fruit During Pregnancy, Does it Protect Against Small for Gestational Age?,” BMC Pregnancy and Childbirth 18 (2018): 486. 30537936 10.1186/s12884-018-2123-4 PMC6288906

[8] N. N. Jusoh and T. A. Tengku Ismail , “A Review of Dietary Intake During Postpartum Period,” IIUM Medical Journal Malaysia 21, no. 1 (2022): 1943.

[9] A. Nikkhah , “A Guide to Nutrition During Antetatal and Post‐Natal Care,” Maternal Pediatric Nutrition 8, no. 2 (2023): 196.

[10] N. E. Marshall , B. Abrams , L. A. Barbour , et al., “The Importance of Nutrition in Pregnancy and Lactation: Lifelong Consequences,” American Journal of Obstetrics and Gynecology 226, no. 5 (2022): 607–632. 34968458 10.1016/j.ajog.2021.12.035 PMC9182711

[11] D. B. Faria‐Schützer , F. G. Surita , L. Rodrigues , and E. R. Turato , “Eating Behaviors in Postpartum: A Qualitative Study of Women With Obesity,” Nutrients 10, no. 7 (2018): 885. 29996489 10.3390/nu10070885 PMC6073558

[12] L. Malek , W. Umberger , M. Makrides , and S. J. Zhou , “Adherence to the Australian Dietary Guidelines During Pregnancy: Evidence From a National Study,” Public Health Nutrition 19, no. 7 (2016): 1155–1163. 26228526 10.1017/S1368980015002232 PMC10271122

[13] J. A. Kavle and M. Landry , “Addressing Barriers to Maternal Nutrition in Low‐and Middle‐Income Countries: A Review of the Evidence and Programme Implications,” Maternal & Child Nutrition 14, no. 1 (2018): e12508. 28836343 10.1111/mcn.12508 PMC5763330

[14] N. R. Reyes , A. A. Klotz , and S. J. Herring , “A Qualitative Study of Motivators and Barriers to Healthy Eating in Pregnancy for Low‐Income, Overweight, African‐American Mothers,” Journal of the Academy of Nutrition and Dietetics 113, no. 9 (2013): 1175–1181. 23871106 10.1016/j.jand.2013.05.014 PMC3782301

[15] R. Stevens , E. Kelaiditi , and K. Myrissa , “Exploration of the Dietary Habits, Lifestyle Patterns and Barriers to Healthy Eating in UK Post‐Partum Women,” Nutrition Bulletin 46, no. 1 (2021): 26–39.

[16] J. A. McNamara , N. Z. Mena , A. Wright , and M. L. Barr , “There's a lot of Like, Contradicting Stuff—Views on Healthy Living During Pregnancy and Postpartum,” International Journal of Environmental Research and Public Health 19, no. 10 (2022): 5849. 35627385 10.3390/ijerph19105849 PMC9140655

[17] M. C. Kay , M. Bentley , and H. Wasser , “Barriers and Facilitators to Healthy Eating During Post‐Partum Among Non‐Hispanic Black Mothers,” Maternal & Child Nutrition 21 (2024): e13741. 39392194 10.1111/mcn.13741 PMC11650027

[18] L. N. Grenier , S. A. Atkinson , M. F. Mottola , et al., “Be Healthy in Pregnancy: Exploring Factors That Impact Pregnant Women's Nutrition and Exercise Behaviours,” Maternal & Child Nutrition 17, no. 1 (2021): e13068. 32705811 10.1111/mcn.13068 PMC7729656

[19] T. C. J. Burton , N. Crooks , L. Pezley , et al., “Food Choice and Dietary Perspectives of Young, Urban, Black Pregnant Women: A Focus Group Study,” Nutrients 16, no. 6 (2024): 781. 38542692 10.3390/nu16060781 PMC10974382

[20] G. Jeong , S. W. Park , Y. K. Lee , S. Y. Ko , and S. M. Shin , “Maternal Food Restrictions During Breastfeeding,” Korean Journal of Pediatrics 60, no. 3 (2017): 70. 28392822 10.3345/kjp.2017.60.3.70 PMC5383635

[21] B. R. Olajide , P. van der Pligt , and F. H. McKay , “Cultural Food Practices and Sources of Nutrition Information Among Pregnant and Postpartum Migrant Women From Low‐ and Middle‐Income Countries Residing in High Income Countries: A Systematic Review,” PLoS One 19, no. 5 (2024): e0303185. 38723007 10.1371/journal.pone.0303185 PMC11081330

[22] S. Jayasinghe , N. M. Byrne , and A. P. Hills , “Cultural Influences on Dietary Choices,” Progress in Cardiovascular Diseases 90 (2025): 22–26. 39921186 10.1016/j.pcad.2025.02.003

[23] M. Ramulondi , H. de Wet, , and N. R. Ntuli , “Traditional Food Taboos and Practices During Pregnancy, Postpartum Recovery, and Infant Care of Zulu Women in Northern KwaZulu‐Natal,” Journal of Ethnobiology and Ethnomedicine 17, no. 1 (2021): 15. 33743760 10.1186/s13002-021-00451-2 PMC7981893

[24] H. Gebregziabher , A. Kahsay , F. Gebrearegay , K. Berhe , A. Gebremariam , and G. G. Gebretsadik , “Food Taboos and Their Perceived Reasons Among Pregnant Women in Ethiopia: A Systematic Review, 2022,” BMC Pregnancy and Childbirth 23, no. 1 (2023): 116. 36797675 10.1186/s12884-023-05437-4 PMC9933406

[25] Y. Agus , S. Horiuchi , and S. E. Porter , “Rural Indonesia Women's Traditional Beliefs About Antenatal Care,” BMC Research Notes 5, no. 1 (2012): 589. 23106915 10.1186/1756-0500-5-589 PMC3532090

[26] M. A. Dalaba , E. A. Nonterah , S. T. Chatio , et al., “Culture and Community Perceptions on Diet for Maternal and Child Health: A Qualitative Study in Rural Northern Ghana,” BMC Nutrition 7 (2021): 36. 34261513 10.1186/s40795-021-00439-x PMC8281629

[27] M. Quintanilha , M. J. Mayan , J. Thompson , and R. C. Bell , “Contrasting “Back Home” and “Here”: How Northeast African Migrant Women Perceive and Experience Health During Pregnancy and Postpartum in Canada,” International Journal for Equity in Health 15 (2016): 80. 27225663 10.1186/s12939-016-0369-x PMC4881207

[28] F. Iradukunda , K. M. Harper , M. T. Paterno , and K. Poudel‐Tandukar , “Dietary Transition Among Sub‐Saharan Africa Women Post‐Immigration and During Pregnancy,” Ethnicity & Health 27, no. 6 (2022): 1329–1344. 33565334 10.1080/13557858.2021.1879027

[29] F. Iradukunda and K. Poudel‐Tandukar , “Healthy Diet Perceptions of Pregnant Women From Sub‐Saharan Africa Residing in the US,” Ecology of Food and Nutrition 60, no. 6 (2021): 682–696. 33467928 10.1080/03670244.2021.1875457

[30] Federation of Ethnic Communities' Councils of Australia (FECCA) ., “AUSTRALIAN MOSAIC.” The magazine of the Federation of Ethnic Communities' Councils of Australia (FECCA, 2017).

[31] Australian Bureau of Statistics ., Australia's Population by Country of Birth [Internet] (Canberra: ABS, 2023 June).

[32] M. Eslier , E. Azria , K. Chatzistergiou , Z. Stewart , A. Dechartres , and C. Deneux‐Tharaux , “Association Between Migration and Severe Maternal Outcomes in High‐Income Countries: Systematic Review and Meta‐Analysis,” PLoS Medicine 20, no. 6 (2023): e1004257. 37347797 10.1371/journal.pmed.1004257 PMC10328365

[33] F. B. Belihu , M.‐A. Davey , and R. Small , “Perinatal Health Outcomes of East African Immigrant Populations in Victoria, Australia: A Population Based Study,” BMC Pregnancy and Childbirth 16, no. 1 (2016): 86. 27113930 10.1186/s12884-016-0886-z PMC4845379

[34] X. Glaw , K. Inder , A. Kable , and M. Hazelton , “Visual Methodologies in Qualitative Research:Autophotography and Photo Elicitation Applied to Mental Health Research,” International Journal of Qualitative Methods 16, no. 1 (2017): 1609406917748215.

[35] V. M. Richard and L. Mke , “Photo‐Elicitation: Reflexivity on Method, Analysis, and Graphic Portraits,” International Journal of Research & Method in Education 38, no. 1 (2015): 3–22.

[36] D. Harper , “Talking About Pictures: A Case for Photo Elicitation,” Visual Studies 17, no. 1 (2002): 13–26.

[37] B. R. Olajide , P. van der Pligt, , V. Vasilevski , et al., “Cultural Food Practices During Pregnancy and the Postpartum Period Among African Migrant Women Living in Australia: A Qualitative Study,” Journal of Racial and Ethnic Health Disparities (2025): 1–12, 10.1007/s40615-025-02690-5. 41062847

[38] J. M. Morse , “Data Were Saturated,” Qualitative Health Research 25, no. 5 (2015): 587–588. 25829508 10.1177/1049732315576699

[39] L. Birt , S. Scott , D. Cavers , C. Campbell , and F. Walter , “Member Checking: A Tool to Enhance Trustworthiness or Merely a Nod to Validation?,” Qualitative Health Research 26, no. 13 (2016): 1802–1811. 27340178 10.1177/1049732316654870

[40] J. Green and N. Thorogood , Qualitative Methods for Health Research (SAGE Publications, 2018). 2018, Fourth ed, 420.

[41] B. C. O'Brien , I. B. Harris , T. J. Beckman , D. A. Reed , and D. A. Cook , “Standards for Reporting Qualitative Research: A Synthesis of Recommendations,” Academic Medicine 89, no. 9 (2014): 1245–1251. 24979285 10.1097/ACM.0000000000000388

[42] S. W. Groth , A. H. Simpson , and I. D. Fernandez , “The Dietary Choices of Women who are Low‐Income, Pregnant, and African American,” Journal of Midwifery & Women's Health 61, no. 5 (2016): 606–612. 10.1111/jmwh.12463 27448099

[43] U. Ekwochi , C. D. I. Osuorah , I. K. Ndu , C. Ifediora , I. N. Asinobi , and C. B. Eke , “Food Taboos and Myths in South Eastern Nigeria: The Belief and Practice of Mothers in the Region,” Journal of Ethnobiology and Ethnomedicine 12 (2016): 7. 26818243 10.1186/s13002-016-0079-x PMC4729178

[44] B. K. Maykondo , C. Horwood , L. Haskins , et al., “A Qualitative Study to Explore Dietary Knowledge, Beliefs, and Practices Among Pregnant Women in a Rural Health Zone in the Democratic Republic of Congo,” Journal of Health, Population, and Nutrition 41, no. 1 (2022): 51. 36414967 10.1186/s41043-022-00333-7 PMC9682828

[45] R. Raghavan , C. Dreibelbis , B. L. Kingshipp , et al., “Dietary Patterns Before and During Pregnancy and Maternal Outcomes: A Systematic Review,” American Journal of Clinical Nutrition 109 (2019): 705S–728SS. 30982868 10.1093/ajcn/nqy216

[46] M. J. Netting , P. F. Middleton , and M. Makrides , “Does Maternal Diet During Pregnancy and Lactation Affect Outcomes in Offspring? A Systematic Review of Food‐Based Approaches,” Nutrition 30, no. 11–12 (2014): 1225–1241. 25280403 10.1016/j.nut.2014.02.015

[47] A. Imdad and Z. A. Bhutta , “Effect of Balanced Protein Energy Supplementation During Pregnancy on Birth Outcomes,” BMC Public Health 11 (2011): 1–9. 21501434 10.1186/1471-2458-11-S3-S17 PMC3231890

[48] R. Baskin , B. Hill , F. N. Jacka , A. O'neil , and H. Skouteris , “Antenatal Dietary Patterns and Depressive Symptoms During Pregnancy and Early Post‐Partum,” Maternal & Child Nutrition 13, no. 1 (2017): e12218. 26725347 10.1111/mcn.12218 PMC6866222

[49] M. L. Kearns and C. M. Reynolds , “The Impact of Non‐Nutritive Sweeteners on Fertility, Maternal and Child Health Outcomes—A Review of Human and Animal Studies,” Proceedings of the Nutrition Society 83 (2024): 280–292. 10.1017/S0029665124000168 38433591

[50] C. Cai , A. Sivak , and M. H. Davenport , “Effects of Prenatal Artificial Sweeteners Consumption on Birth Outcomes: A Systematic Review and Meta‐Analysis,” Public Health Nutrition 24, no. 15 (2021): 5024–5033. 33441213 10.1017/S1368980021000173 PMC11082813

[51] R. Blanchet , C. P. Nana , D. Sanou , M. Batal , and I. Giroux , “Dietary Acculturation Among Black Immigrant Families Living in Ottawa—A Qualitative Study,” Ecology of Food and Nutrition 57, no. 3 (2018): 223–245. 29617162 10.1080/03670244.2018.1455674

[52] L. Ngongalah , T. Rapley , J. Rankin , and N. Heslehurst , “Cultural Influences on African Migrant Pregnant and Postnatal Women's Dietary Behaviours and Nutrition Support Needs in the UK,” Nutrients 15, no. 19 (2023): 4135. 37836419 10.3390/nu15194135 PMC10574463

[53] D. L. Olstad and L. McIntyre , “Educational Attainment as a Super Determinant of Diet Quality and Dietary Inequities,” Advances in Nutrition 16, no. 9 (2025): 100482. 10.1016/j.advnut.2025.100482 PMC12361756 40683372

[54] J. Lê , J. Dallongeville , A. Wagner , et al., “Attitudes Toward Healthy Eating: A Mediator of the Educational Level–Diet Relationship,” European Journal of Clinical Nutrition 67, no. 8 (2013): 808–814. 23801096 10.1038/ejcn.2013.110

[55] A. C. Maia , M. J. Marques , A. R. Goes , A. Gama , R. Osborne , and S. Dias , “Health Literacy Strengths and Needs Among Migrant Communities From Portuguese‐Speaking African Countries in Portugal: A Cross‐Sectional Study,” Frontiers in Public Health 12 (2024): 1415588. 39022410 10.3389/fpubh.2024.1415588 PMC11253791

