# Supplementary material for: Perceptions of African Migrant Women Regarding Food Consumption During Pregnancy and the Postpartum Period in Australia: A Qualitative Study
Source: J Hum Nutr Diet. 2026 Mar 29;39(2):e70237. doi: 10.1111/jhn.70237 (PMC13033822; doi:10.1111/jhn.70237)
Supplement: Supplementary file 1 — Supporting file 1. [file JHN-39-0-s003.docx]

**Supplementary file 1. Photo-elicitation Interview Guide**

Semi-structured questions used to explore women’s perceptions of healthy and unhealthy foods during pregnancy and the postpartum period.

*I want to sincerely thank you for taking the time to participate in this second interview. This interview aims to gain a deeper understanding of the food practices of African migrant women during pregnancy. This is an opportunity for you to express yourself more and reflect on the pictures you took.*

*I believe that you have read and consented to participate in this research, but I would like you to go through the consent form again and check you are happy to be part of the research.*

*There are no right or wrong answers, and your opinions, ideas, and responses are valuable. Inform me of any queries, breaks, or if you wish to terminate the interview.*

*Do you have any questions before we begin?*

**Tell me about these photos**

1. The names of each food in the pictures
2. Does this reflect your usual food intake when you are not pregnant? If not, why did you adopt these food practices during pregnancy?
3. In your opinion, why do you think these foods are healthy (or unhealthy) for consumption during pregnancy or postpartum? Where did you find out about these foods being healthy?
4. Why should these foods be eaten during pregnancy?

*(Prompts: To protect the mother’s health, fetus's health, prevent pregnancy complications, for baby’s beautiful appearance after birth?)*

1. Why should these foods be avoided during pregnancy

*(Prompts: avoid giving birth to a large baby, causes miscarriage?)*

1. Why should these foods be eaten during postpartum?
2. Why should these foods be avoided during postpartum?
3. Do these foods differ from what your mother or grandmother may have believed to be a healthy food choice? Why?
