# Supplementary material for: Perceptions of African Migrant Women Regarding Food Consumption During Pregnancy and the Postpartum Period in Australia: A Qualitative Study
Source: J Hum Nutr Diet. 2026 Mar 29;39(2):e70237. doi: 10.1111/jhn.70237 (PMC13033822; doi:10.1111/jhn.70237)
Supplement: Supplementary file 3 — Supporting file 3. [file JHN-39-0-s001.docx]

**Supplementary file 3. Participant-provided photographs illustrating perceived healthy and unhealthy foods**

This file contains photographs of foods submitted by participants to illustrate items they perceived as healthy or unhealthy during pregnancy and the postpartum period. The photographs were used to prompt discussion during the interviews.


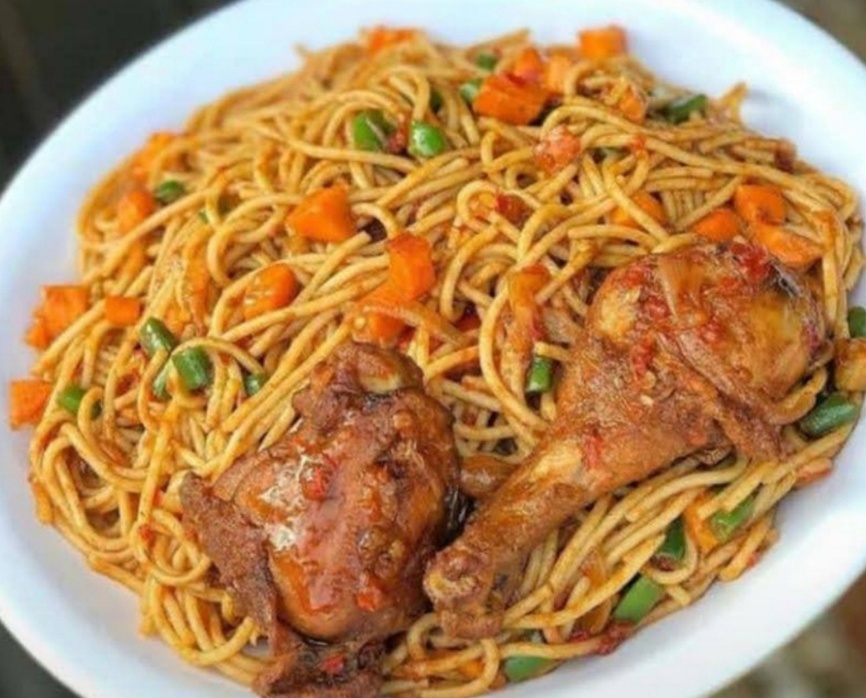


Spaghetti with vegetables and chicken, photographed by a participant to illustrate their perception of a balanced meal


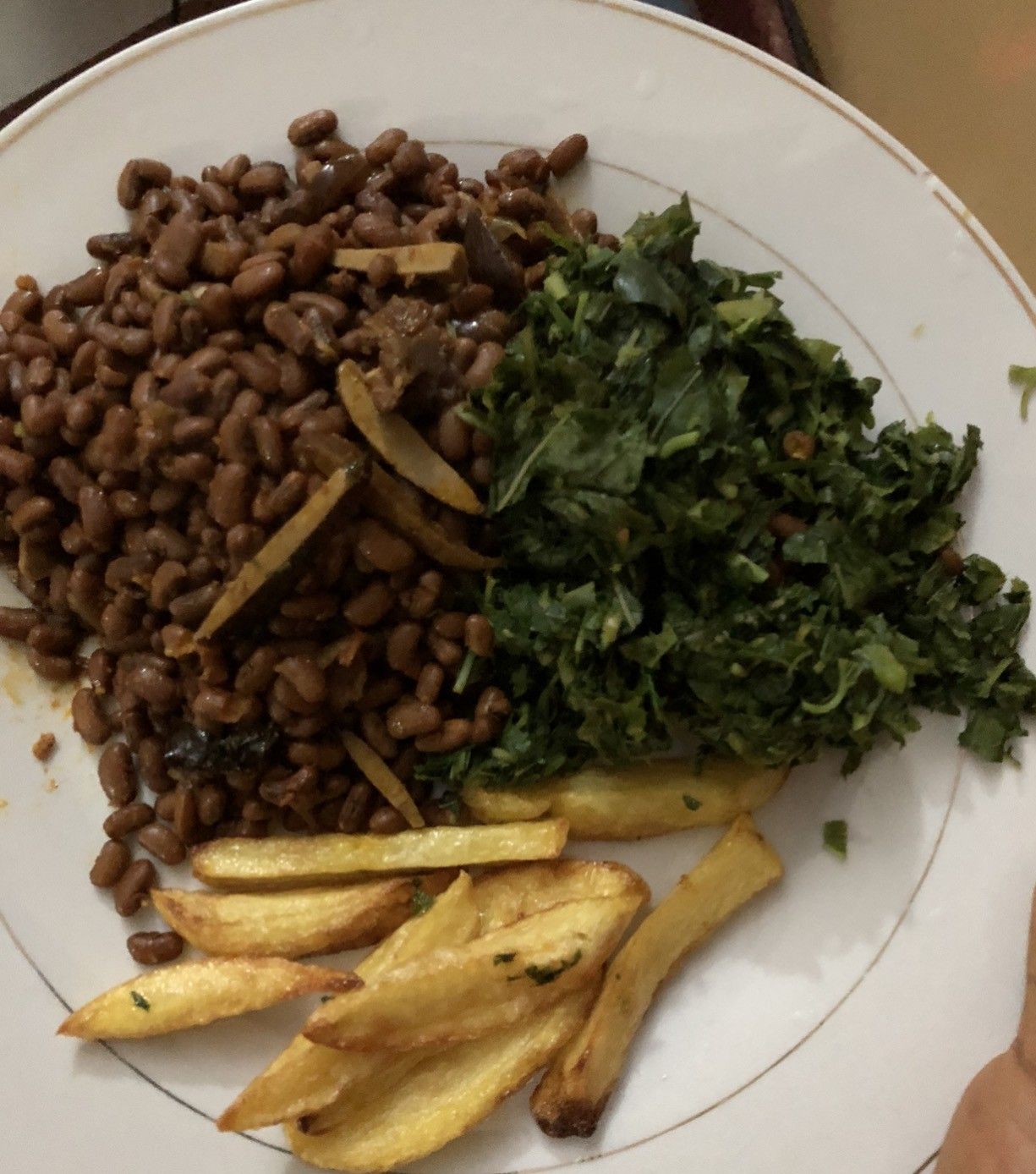


Brown bean with Irish potatoes and amaranthus (vegetable), photographed by a participant to depict a homemade meal they consider healthy


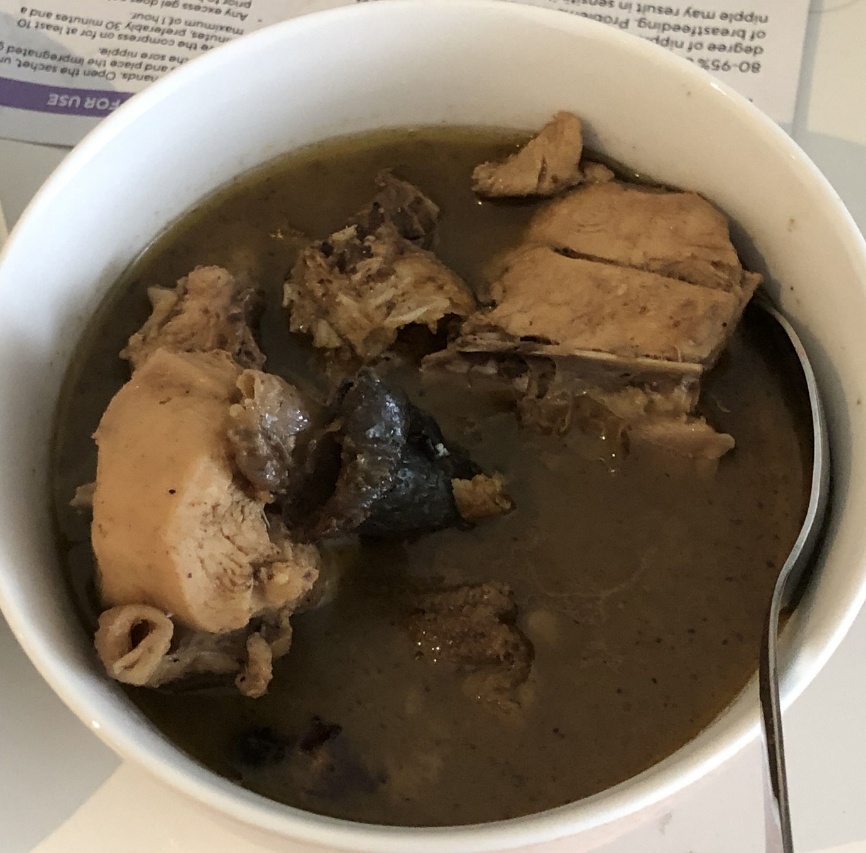


Traditional soup (chicken and dried fish), photographed by a participant to depict a nutritious meal consumed during the postpartum period, perceived to support breastfeeding and recovery after childbirth


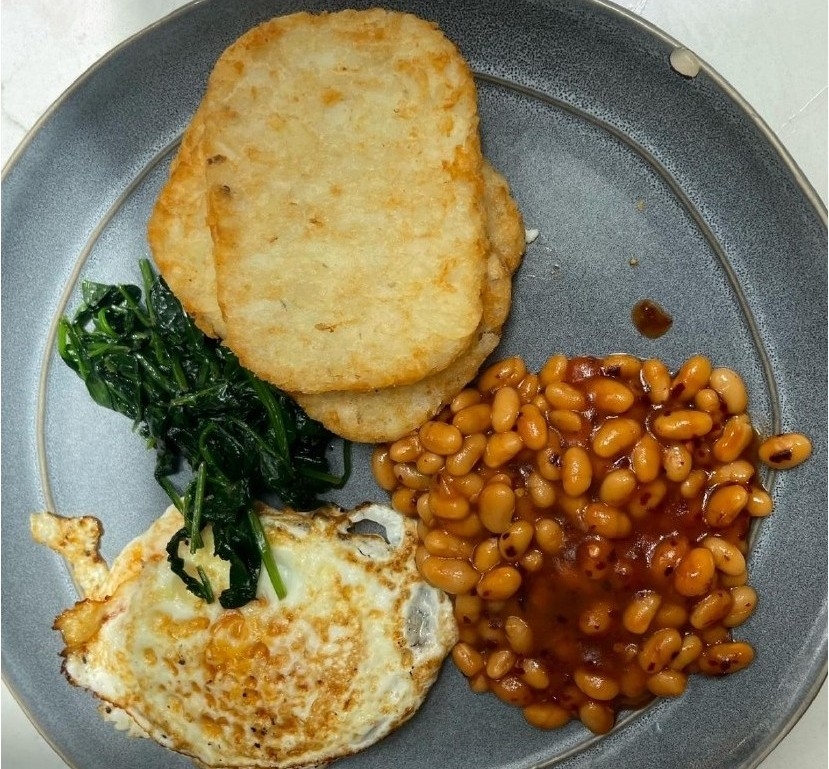


Eggs, spinach, hash brown (on the left side of the plate), and baked beans (on the right side of the plate). Photographed by a participant to depict a meal they considered unhealthy, particularly due to the inclusion of hash brown and baked beans


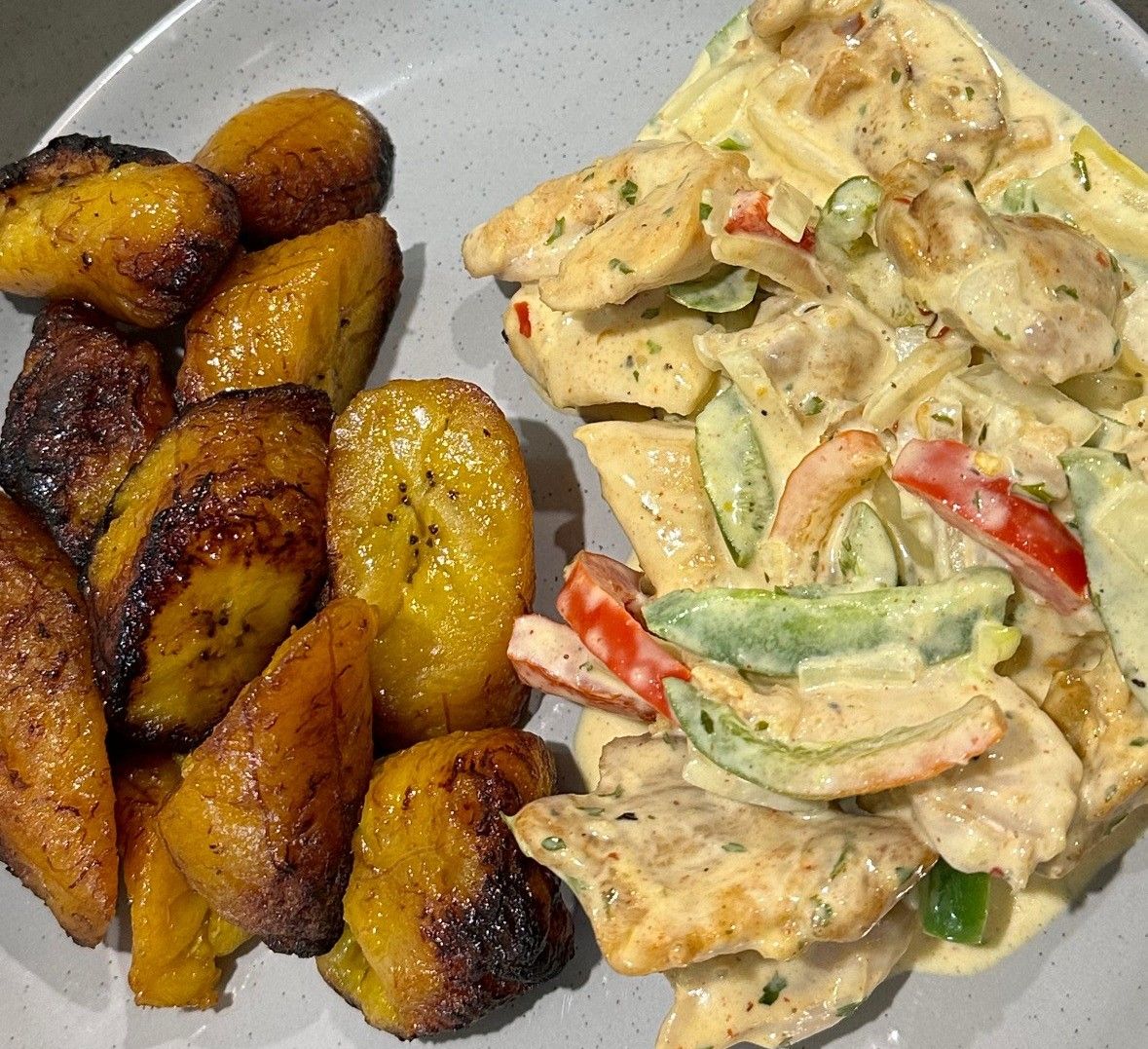


Fired plantain and chicken mayonnaise, photographed by a participant to depict foods they considered unhealthy
